# P-562. Tracking the Increase: Diagnostic Trends in Infectious Disease Among New Patients at a Large Urban Safety Net Health System, New York City (NYC) 2021-2023

**DOI:** 10.1093/ofid/ofaf695.777

**Published:** 2026-01-11

**Authors:** Eliana Jacobson, Zeyu Li, Remle Newton-Dame, Coral Naomi Vargas-Pena, Maurice Policar, Christina Coyle, Justin Chan

**Affiliations:** NYU Langone, New York, NY; NYC Health + Hospitals, New York, New York; NYC Health + Hospitals, New York, New York; Albert Einstein College of Medicina/Jacobi Medical Center, Bronx, New York; Health + Hospitals / Elmhurst, Elmhurst, New York; NYC Health + Hospitals/Jacobi, Albert Einstein College of Medicine, New York, New York; NYC Health + Hospitals, New York, New York

## Abstract

**Background:**

NYC Health + Hospitals (H+H), the nation’s largest municipal health system, provides healthcare to a large proportion of uninsured and socially vulnerable persons, including those newly arrived to the city. Diagnostic trends among new H+H patients can reveal insights in emerging demands on primary and specialty healthcare services. We aim to describe recent trends in infectious disease diagnoses among patients who recently initiated care at H+H.

**Table 1:**
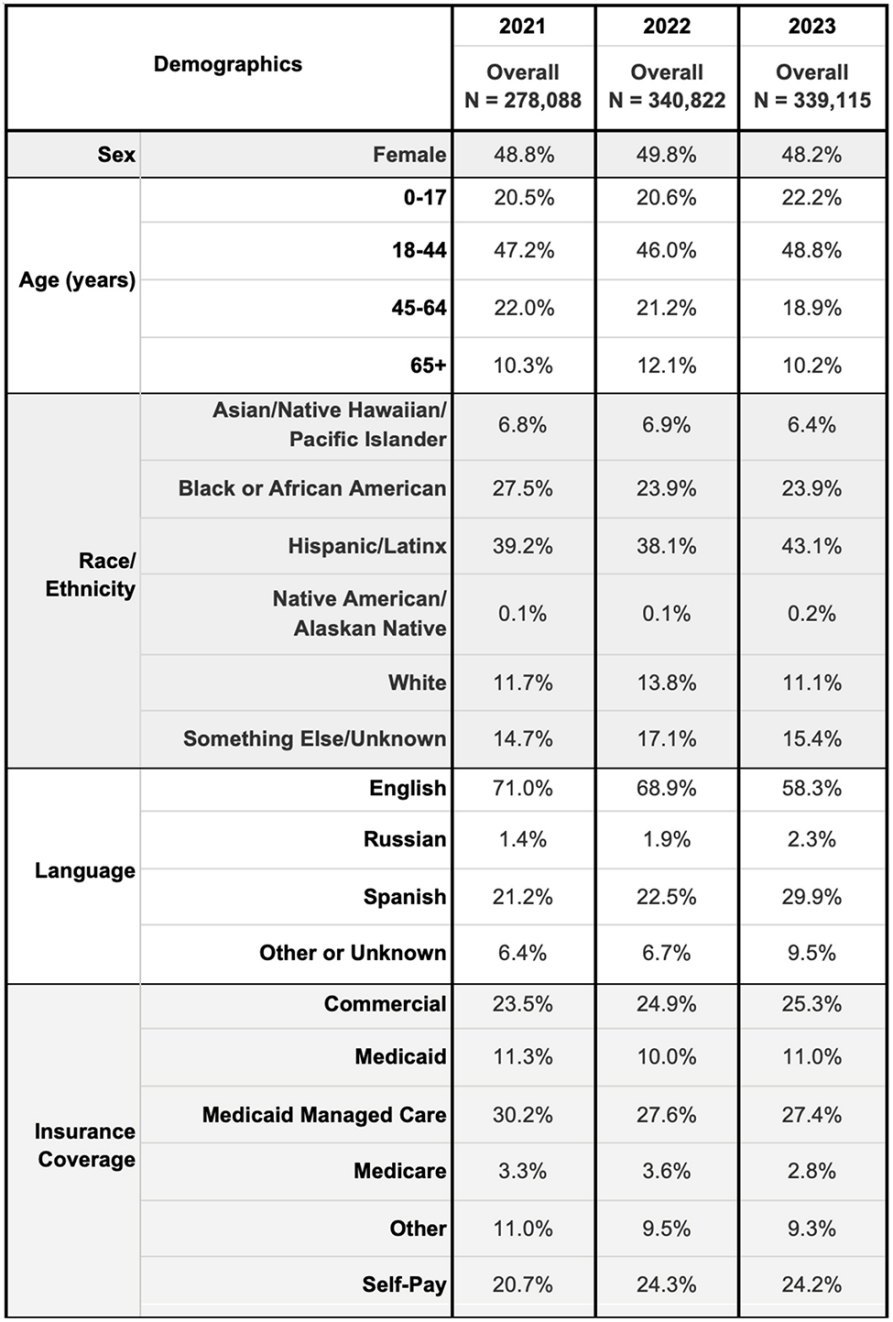
Characteristics of annual cohorts of patients new to NYC Health + Hospitals, New York City 2021-2023

**Table 2a:**
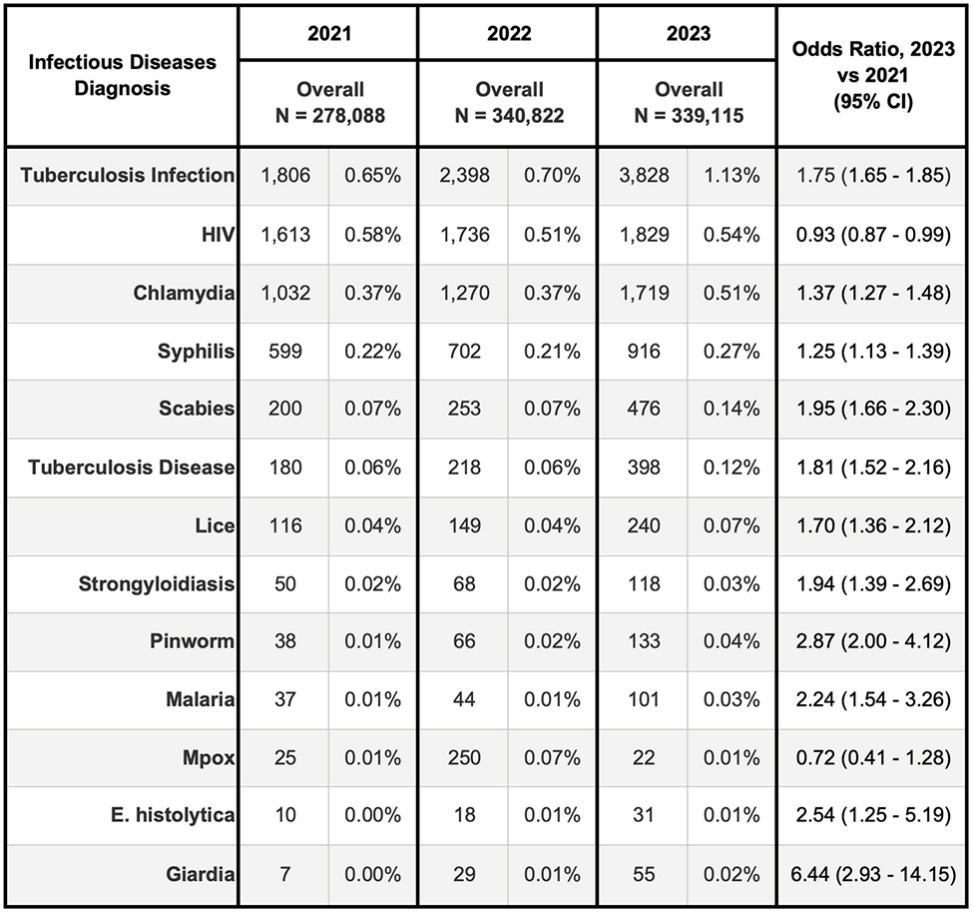
Frequency of infectious diseases diagnoses (by ICD-10-CM codes) made in the 12 months after new engagement with NYC Health + Hospitals, New York City 2021-2023 Overall cohort

**Methods:**

We included three cohorts of patients with first-time visits to H+H in 2021, 2022, or 2023, respectively. Index medical visits were identified using 2017-2023 records from H+H’s electronic health record (EHR). We identified patients with a subset of infectious disease diagnoses, using International Classification of Diseases, Tenth Revision, Clinical Modification (ICD-10-CM) codes, including EHR billing and visit diagnoses, within 12 months of each patient’s index visit. We compared patient characteristics and odds ratios of infectious diseases diagnoses between the 2023 and 2021 cohorts.

**Table 2b:**
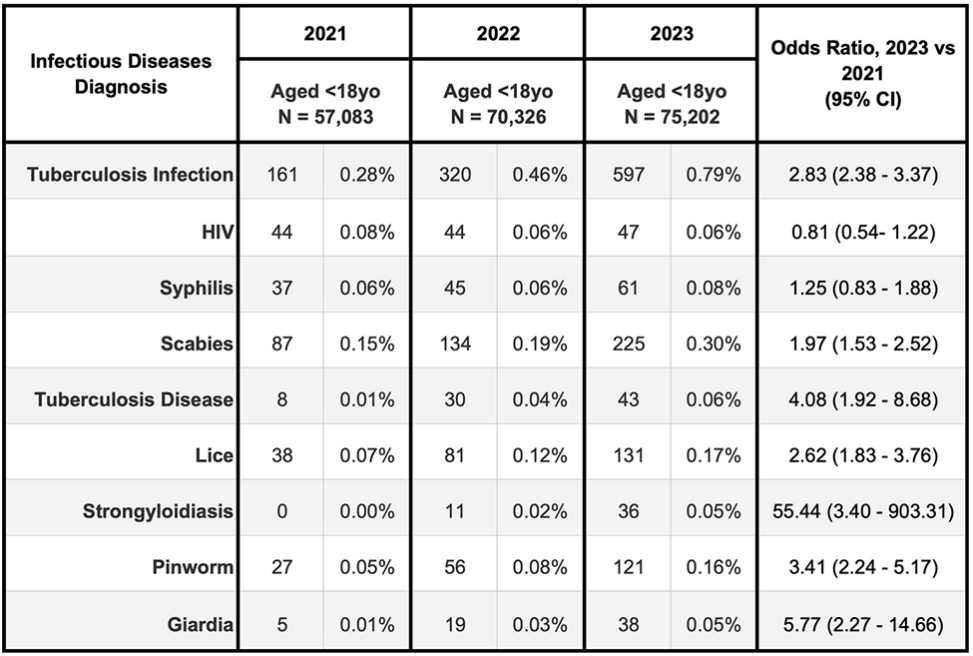
Frequency of infectious diseases diagnoses (by ICD-10-CM codes) made in the 12 months after new engagement with NYC Health + Hospitals, New York City 2021-2023 Cohort aged <18 year-old

**Table 2c:**
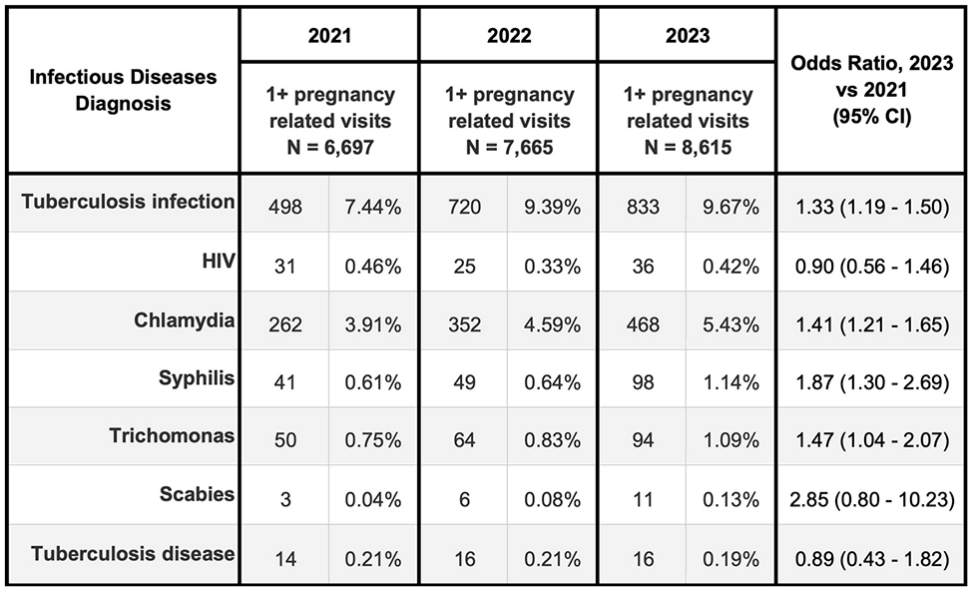
Frequency of infectious diseases diagnoses (by ICD-10-CM codes) made in the 12 months after new engagement with NYC Health + Hospitals, New York City 2021-2023 Cohort with at least 1 pregnancy-related visit

**Results:**

H+H saw 339,115 new patients in 2023 versus 278,088 in 2021 (22% increase), with growth in Spanish speaking and self-pay patients (Table 1). Several infectious disease diagnoses rose disproportionately from 2021 to 2023, including tuberculosis infection (Odds Ratio: 1.8) and disease (1.8), strongyloidiasis (1.9), and malaria (2.2) (Table 2a). Among those aged < 18 years, we saw a large increase for tuberculosis infection (2.8) and disease (4.1), lice (2.6), and pinworm (3.4) diagnoses (Table 2b). Among those who had pregnancy-related visits, diagnoses increased for tuberculosis infection (1.3), chlamydia (1.4), syphilis (1.9), and trichomonas (1.5) (Table 2c).

**Conclusion:**

H+H has seen an increase in several important infectious diseases diagnoses among patients newly encountered in 2021-2023. Our study methodology cannot distinguish between increased prevalence and increased surveillance. However, these increases in diagnosis frequency suggest the need for sustained investment in infectious diseases and related services, including microbiology, parasitology, infection control, epidemiology and public health, to adequately serve the H+H patient population.

**Disclosures:**

All Authors: No reported disclosures

